# Evolution of Blood Innate Immune Cell Phenotypes Following SARS-CoV-2 Infection in Hospitalized Patients with COVID-19

**DOI:** 10.3390/cells14141093

**Published:** 2025-07-17

**Authors:** Arnaud Dendooven, Stephane Esnault, Marie Jacob, Jacques Trauet, Emeline Delaunay, Thomas Guerrier, Amali E. Samarasinghe, Floriane Mirgot, Fanny Vuotto, Karine Faure, Julien Poissy, Marc Lambert, Myriam Labalette, Guillaume Lefèvre, Julie Demaret

**Affiliations:** 1 Univ. Lille, INSERM, CHU Lille, U1286-INFINITE-Institute for Translational Research in Inflammation, F-59000 Lille, France; marie1.jacob@chu-lille.fr (M.J.); jacques.trauet@chu-lille.fr (J.T.); emeline.delaunay@chu-lille.fr (E.D.); thomas.guerrier@chru-lille.fr (T.G.); myriam.labalette@chu-lille.fr (M.L.); guillaume.lefevre@chu-lille.fr (G.L.); julie.demaret@chu-lille.fr (J.D.); 2 CHU Lille, Institut d’Immunologie, F-59000 Lille, France; floriane.casat@chu-lille.fr; 3 Division of Allergy, Pulmonary and Critical Care Medicine, Department of Medicine, University of Wisconsin-Madison School of Medicine and Public Health, Madison, WI 53705, USA; amali.samarasinghe@wisc.edu; 4 CHU Lille, Service Universitaire de Maladies Infectieuses, F-59000 Lille, France; fanny.vuotto@chu-lille.fr (F.V.); karine.faure@chu-lille.fr (K.F.); 5 Univ. Lille, U1019-UMR 9017-CIIL-Center for Infection and Immunity of Lille, F-59000 Lille, France; 6 CHU Lille, Pôle de Réanimation CNRS Inserm U1285-UMR 8576-UGSF-Unité de Glycobiologie, Structurale et Fonctionnelle Univ. Lille, F-59000 Lille, France; julien.poissy@chu-lille.fr; 7 CHU Lille, Service de Médecine Interne et Immunologie Clinique, F-59000 Lille, France; marc.lambert@chu-lille.fr; 8 CHU Lille, Département de Médecine Polyvalente Post-Urgences, F-59000 Lille, France; 9 CEREO, National Reference Center for Hypereosinophilic Syndromes, F-59000 Lille, France

**Keywords:** eosinophils, neutrophils, monocytes, SARS-CoV-2, COVID-19, phenotype, flow cytometry, serum, cytokines, multiplex assay

## Abstract

Innate immune cells appear to have an important implication in the resolution and/or the aggravation of the COVID-19 pathogenesis after infection with SARS-CoV-2. To better appreciate the role of these cells during COVID-19, changes in blood eosinophil, the neutrophil and monocyte count, and levels of surface protein markers have been reported. However, analyses at several timepoints of multiple surface markers on granulocytes and monocytes over a period of one month after a SARS-CoV-2 infection are missing. Therefore, in this study, we performed blood eosinophil, neutrophil, and monocyte phenotyping using a list of surface proteins and flow cytometry during a period of 30 days after the hospitalization of patients with severe SARS-CoV-2 infections. Blood cell counts were reported at seven different timepoints over the 30-day period as well as measures of multiple mediators in serum using a targeted multiplex assay approach. Our results indicate a 95% drop in the blood eosinophil count by D1, with eosinophils displaying a phenotype defined as CD69/CD63/CD125^high^ and CCR3/CD44^low^ during the early phases of hospitalization. Conversely, by D7 the neutrophil count increased significantly and displayed an immature, activated, and immunosuppressive phenotype (i.e., 3% of CD10/CD16^low^ and CD10^low^CD177^high^, 6.7% of CD11b^high^CD62L^low^, and 1.6% of CD16^high^CD62L^low^), corroborated by enhanced serum proteins that are markers of neutrophil activation. Finally, our results suggest a rapid recruitment of non-classical monocytes leaving CD163/CD64^high^ and CD32^low^ monocytes in circulation during the very early phase. In conclusion, our study reveals potential very early roles for eosinophils and monocytes in the pathogenesis of COVID-19 with a likely reprogramming of eosinophils in the bone marrow. The exact roles of the pro-inflammatory neutrophils and the functions of the eosinophils and the monocytes, as well as these innate immune cell types, interplays need to be further investigated.

## 1. Introduction

The SARS-CoV-2 infection is accompanied by changes in the blood innate immune cell numbers and phenotypes, suggesting a possible implication of these immune cells on the severity or resolution of coronavirus disease-2019 (COVID-19). Eosinopenia is a common characteristic of patients with COVID-19, although a subset of patients with severe COVID-19 displayed blood eosinophil numbers equal to eosinophil numbers in the healthy population [1,2,3,4,5]. Phenotypically, during COVID-19, blood eosinophils display increased CD11b (integrin alpha-M) and decreased CD62L (L-selectin), two surface proteins involved in the attachment and rolling on endothelial cells, respectively [5,6]. This eosinophil phenotype (CD11b^high^CD62L^low^) along with eosinopenia in COVID-19 are associated with refractoriness in response to a formyl peptide, which suggests that eosinophils have an activated phenotype, and eosinophils with a full capacity to migrate have already moved from blood to tissue [6]. Conversely, another study found increased amounts of surface CD62L on blood eosinophils from patients with moderate or severe COVID-19 compared to healthy individuals [4]. In that same study, the percentage of CD69^high^ or activated blood eosinophils is higher in COVID-19 [4]. Also, surface CRTH2 (prostaglandin DP2 receptor), a pro-migratory marker, is reduced in COVID-19 in particularly severe COVID-19 [3], suggesting again that eosinopenia was due to the migration to tissue.

While controversial [7], conversely to eosinopenia, increased blood neutrophil numbers are observed in patients with COVID-19, and increased absolute neutrophil counts have been associated with more severe presentations of COVID-19 [3,4,8,9]. In COVID-19, circulating neutrophil subsets varying in their maturity and expression of surface markers have been reported, including increased numbers of immature neutrophils released from bone marrow, which correlate with an increased COVID-19 disease severity [10]. These immature neutrophils were characterized by elevated CD66b and CD24 and lower CD11b, CD177, CXCR1/2, and CD62L compared to mature neutrophils [4]. In another study, severe COVID-19 patients displayed blood neutrophils with reduced surface CD10 and CD62L and increased CD11b compared to healthy participants, indicating an enhanced immaturity and activation state [5]. Notably, a specific neutrophil subset of CD62L^low^ neutrophils is more prone to form NET-like structures [11,12], which have been found in severe COVID-19 patients and correlates with disease severity and thrombosis [13].

The recruitment of monocytes into lung tissue in COVID-19 is associated with increased blood non-classical (NC) (CD14^dim^CD16^high^) and decreased classical (CL) (CD14^high^CD16^low^) monocytes [14]. In another study, however, patients with a more serious disease trajectory displayed a reduced number of blood NC and CX3CL1 [15], a chemokine attracting CX3CR1+ NC monocytes. In addition, in COVID-19 blood monocytes display an activated phenotype (i.e., CD11b/CD16/CD66b^high^), and CD163/CD206^low^ monocytes are decreased in severe compared to mild COVID-19, also suggesting a more activated phenotype [16].

These previous studies mostly focused on a single timepoint, lacking a longitudinal determination of cell phenotypes that would help to better comprehend changes in the inflammatory innate blood cell populations and their possible role during COVID-19. To address this gap of knowledge, we conducted a longitudinal study with hospitalized patients infected by SARS-CoV-2 to follow their blood cell counts and phenotypes of granulocytes and monocytes over a period of 30 days. Our hypothesis is that these immune cells present different kinetics vis-à-vis the recruitment and activation status during the progression of the disease. To help with the interpretation of the data, we also measured cytokines, chemokines, and markers of the activation of these cells in the serum of patients and compared their levels with levels in sera from healthy individuals. This integrated approach aimed to provide a more complete view of the innate immune response in COVID-19.

## 2. Materials and Methods

### 2.1. The Description of the Study and Participants

Forty adults with a diagnosis of COVID-19, documented by SARS-CoV-2 polymerase chain reaction (PCR), and eighteen healthy individuals were enrolled in this study for the phenotyping of innate immune cells (Table 1). All patients were hospitalized for respiratory symptoms, including cough and/or breathing difficulties (Table 1). The severity was estimated according to the WHO ordinal clinical severity scale, collected during hospitalization. The 9 points of the scale are as follows: 0: no clinical or virological evidence of infection; 1: ambulatory, no activity limitation; 2: ambulatory, activity limitation; 3: hospitalized, no oxygen therapy; 4: hospitalized, oxygen mask or nasal prongs; 5: hospitalized, noninvasive mechanical ventilation or high-flow nasal cannula; 6: hospitalized, intubation and invasive mechanical ventilation (IMV); 7: hospitalized, IMV + additional support such as pressors or extracardiac membranous oxygenation; 8: death [17]. In the present study, all patients have a score ≥ 4, and the retained score corresponded to the most severe situation during hospitalization. For example, if a patient required only oxygen with nasal prongs at admission and subsequently required intubation and invasive mechanical ventilation several days after, the recorded severity score was 6. Blood samples were collected at the University Hospital of Lille prospectively over a 30-day period starting from the first day (D1) of the hospitalization. Blood cell phenotypic determinations were categorized into four timeframes: “very early” (D1 to D3 of hospitalization); “early” (D5 to D9); “mid-point” (D14); and “late” (D30 from D1 of hospitalization). Samples were obtained from August 2020 to November 2020. This study was performed in accordance with the Declaration of Helsinki principles for ethical research. All data were anonymized before analysis. This study was approved by the French data protection agency (CNIL: Commission Nationale de l’informatique et des libertés, registration # DEC20-086) and by the local ethics committee (ID-CRB 2020-A00763-36). Participants were provided with an informed consent form that they signed before enrollment. Control data from healthy subjects were taken from another study looking at the immunogenicity of SARS-CoV-2 vaccine in elderly subjects (NCT04760704). Each healthy subject had signed an informed consent form allowing the reuse of their data for other research purposes.

### 2.2. Blood Collection and Cell Enumeration

Peripheral blood (5 mL) was collected in EDTA (disodium salt of ethylenediaminetetraacetic acid) tubes. Blood cells count and leukocyte subset quantification were performed on the indicated days using a Sysmex XN hematology analyzer (Sysmex, Norderstedt, Germany).

### 2.3. Flow Cytometry

Eosinophil surface markers (CD69, HLA-DR, CCR3, CD125, CRTH2, CD44, and CD63), neutrophil surface markers (CD11b, CD62L, CXCR1, CXCR2, CD10, CD16, and CD32) and monocyte surface markers (CD68, CD206, CD80, CD163, IFNAR1, IFNAR2, HLA-DR, CD16, CD32, and CD64) were analyzed using distinct panels (Supplementary Table S1). Details regarding the analyses of eosinophil phenotyping have been previously reported [18]. Briefly, each panel was incubated separately with 100 µL of whole blood for 10 min in the dark at room temperature. Red blood cells were lysed using the TQ-Prep system (Beckman-Coulter, Brea, CA, USA), followed by two consecutive washes at 1800 rpm for 5 min with 3 mL of phosphate-buffered saline (PBS). The cell pellet was resuspended in 300 µL of PBS before acquisition on a standardized CytoFLEX S Flow Cytometer (Beckman Coulter). Data analysis was performed using Kaluza 2.1 software (Beckman Coulter). Detailed gating strategies for identification of each cell type are provided in Supplementary Figures S1–S3.

### 2.4. Luminex

Serum was collected after centrifugation of whole blood and stored at −80 °C until analyses. In addition to 18 healthy individuals used for cell phenotyping, 13 sera from healthy participants were added for measurements by Luminex (Table 2). Targeted inflammatory mediators were quantified using the Luminex^®^ Discovery Assay (R&D Systems, Minneapolis, MN, USA), following the manufacturer’s instructions. Briefly, analyte-specific microbeads coated with capture antibodies were incubated with serum samples diluted 1:2 in the recommended buffer. Bound analytes were detected using biotinylated detection antibodies and a streptavidin-phycoerythrin conjugate. Fluorescence intensity was measured on a Bioplex 200 System (Bio-Rad, Hercules, CA, USA), ensuring precise and specific quantification of the markers of interest.

### 2.5. Statistics

All data were analyzed using GraphPad Prism (version 10.4.0). For blood cell counts across different timepoints, comparisons were performed using a one-way analysis of variance (ANOVA) followed by a Holm–Sidak post hoc test to correct for multiple comparisons. Non-parametric statistical methods were applied for the analysis of flow cytometry and serum mediator data. Specifically, median fluorescence intensities (MFIs) of eosinophil, neutrophil, and monocyte activation markers at each timepoint were compared to the corresponding MFI of healthy controls using a Kruskal–Wallis test followed by Dunn’s post hoc test. Similarly, the concentrations of inflammatory mediators at each timepoint were compared to those measured in healthy subjects using the same statistical approach. To explore global patterns of variation across timepoints, principal component analyses (PCAs) were performed separately for each immune cell subset. Pairwise correlations between activation markers within each cell type were assessed using Spearman’s rank correlation coefficient. For all statistical tests, a *p*-value < 0.05 was considered significant.

## 3. Results

### 3.1. Participants

The majority of both patients and healthy individuals were males (Table 1). Patients were mostly in their sixties and experienced dyspnea, cough and/or fever, and were treated with corticosteroids, antibiotics, and/or antivirals (Table 1). All patients required oxygen therapy, twenty-six (65%) with noninvasive or invasive mechanical ventilation; five (12.5%) died during their hospitalization. Blood cell phenotypic determinations were categorized into four timeframes: “very early” (D1 to D3 of hospitalization); “early” (D5 to D9); “mid-point” (D14); and “late” (D30 from D1 of hospitalization) (Table 2). For the eosinophil phenotyping by flow cytometry, 13 (antibody panel 1) and 10 (antibody panel 2) patients were analyzed in the “very early” (D1 to D3) phase. Due to severe eosinopenia in the early phase of COVID-19 and technical limitations in multiparametric flow cytometry, the number of patients analyzed for eosinophil markers is lower than for neutrophils or monocytes. When eosinophils were undetectable or below the threshold of reliable detection, the corresponding samples were excluded from eosinophil analyses (flow chart in Supplementary Figure S4). In addition, due to the extended duration of the study and sample collection, and the complexity of multiparametric flow cytometry panels for all cell types, some phenotyping data had to be excluded because of technical issues. Moreover, some patients either died, were discharged from the hospital, did not come back for the blood draw in the following phases, or blood draws were missed at the hospital (Supplementary Figure S4 and Table 2). Neutropenia and monocytopenia were rare in the early phases, and thus the numbers of patients analyzed were higher than for the eosinophils (Table 2). Eosinophil, neutrophil, and monocyte phenotypes were determined in 18 healthy participants at one timepoint (Table 2).

### 3.2. The Blood Cell Count During the Hospitalization

**Figure 1:**
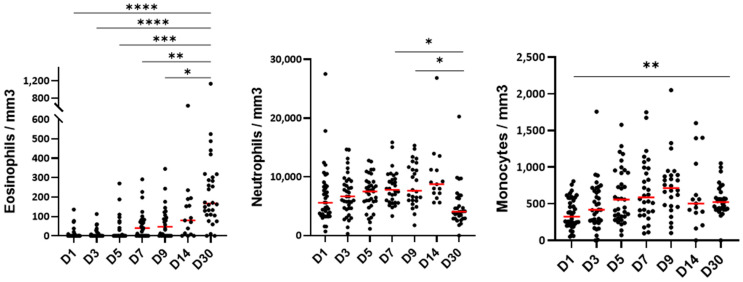
The blood cell count following hospitalization with SARS-CoV-2 infections. The cell count was performed on the indicated days from the first day of hospitalization (D1) until the 30th day (D30). Only the significant differences compared to D30 are shown for the eosinophils. All significant comparisons are shown for the neutrophils and monocytes. Each dot corresponds to one patient and the red lines show the median values. **** *p* < 0.0001, *** *p* < 0.001, ** *p* < 0.01, and * *p* < 0.05. The ANOVA followed by Holm–Sidak’s multiple comparison test.

Blood eosinophil numbers were very low in the first 5 days of hospitalization and gradually increased at D7 to reach normal counts (~200/mm^3^) on day 30 after hospitalization (Figure 1). Conversely to eosinophils, the number of blood neutrophils did not change significantly in the first 5 days of hospitalization, but an augmentation occurred starting on D7 until D14 (Figure 1). Monocyte counts were lower at D1 than at D30 and then progressively increased over time, quickly reaching levels observed at D30 (Figure 1).

### 3.3. Blood Eosinophil Phenotype

**Figure 2:**
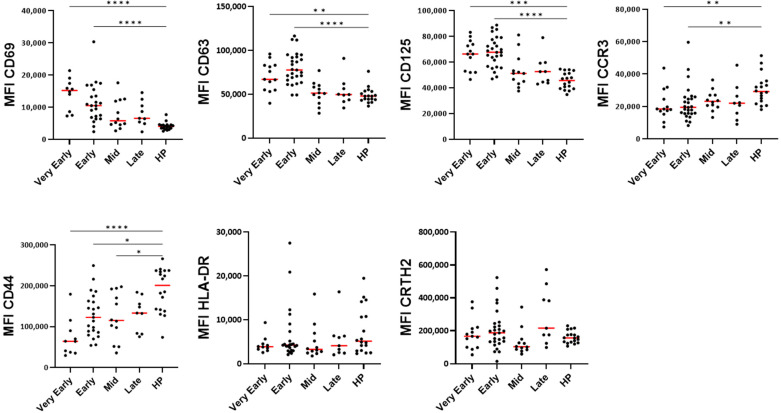
Level of surface proteins on blood eosinophils over time after hospitalization with SARS-CoV-2 infection and compared to healthy controls. As shown in Table 2, timepoints were grouped as “very early” phase (D1 to D3 from hospitalization), “early” phase (D5 to D9), “Mid” phase (D14), and “late” phase (D30). MFI (median fluorescence intensity) for each participant and means are shown. The red lines show the median values. **** *p* < 0.0001, *** *p* < 0.001, ** *p* < 0.01, and * *p* < 0.05. Kruskal–Wallis test with Dunn’s multiple comparisons test.

**Figure 3:**
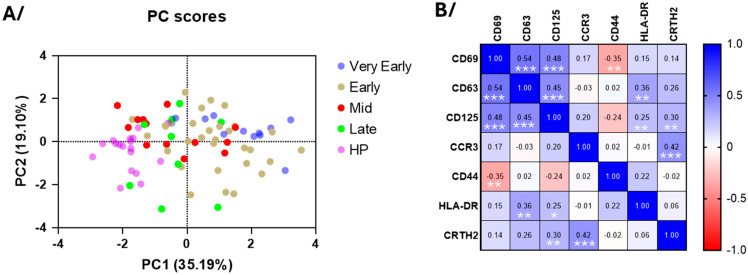
The principle component and correlative analyses of the level of surface membrane proteins on blood eosinophils. (**A**) The principal component analysis of the participants divided as healthy participants (HPs), very early phase, early phase, mid-phase, and late phase COVID-19. PC1 was mostly driven by CD69, CD125, and CD63, and PC2 was mostly driven by CCR3 and CRTH2. (**B**) The correlative analysis (Spearman) of all surface markers for all timepoints in COVID-19 (*n* = 65 to 75). *r* values are indicated, *** *p* < 0.001, ** *p* < 0.01, and * *p* < 0.05.

The major marker of eosinophil activation, CD69, along with CD125 (IL-5 receptor) and CD63 (a marker of secretory activity and degranulation, [19]) were significantly increased for most patients in the very early and early phases after hospitalization compared to healthy individuals (Figure 2). These increases were then followed by normalization at the mid-phase (Figure 2). The CCR3 expression was reduced during most of the course of the disease (Figure 2), suggesting possible interactions with chemokines [20]. The CD44 expression was strongly decreased, whereas HLA-DR and CRTH2 levels did not change (Figure 2). Supporting these results, the principal component analysis (PCA) of eosinophil surface markers confirmed a clear distinction between COVID-19 patients in the early phases and healthy participants (Figure 3A), re-enforcing the distinct eosinophil phenotype during acute COVID-19. PC1 was mainly driven by CD69 (loading = 0.860), CD125 (0.803), and CD63 (0.773), whereas PC2 was mainly driven by CCR3 (0.754) and CRTH2 (−0.757) (Figure 3A), and the correlation analysis demonstrated strong positive associations between the CD69, CD63, and CD125 surface expression (Figure 3B), while CD44 exhibited an inverse correlation with these three markers. Additionally, in agreement with the PCA, an association was observed between CCR3 and CRTH2 (Figure 3B).

### 3.4. Blood Neutrophil Phenotype

**Figure 4:**
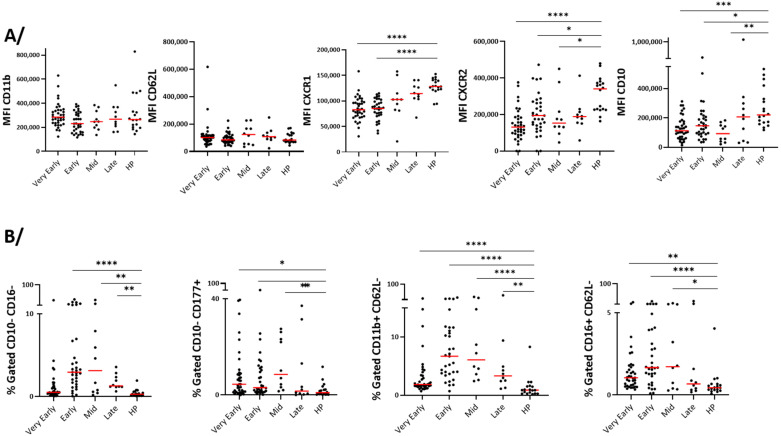
Level of surface proteins on blood neutrophils over time after hospitalization with SARS-CoV-2 infection and compared to healthy controls. As shown in Table 2, timepoints were grouped as “very early” phase (D1 to D3), “early” phase (D5 to D9), “mid” phase (D14), and “late” phase (D30). (**A**) Analysis of one surface marker. (**B**) Analysis of subpopulations using two surface markers. MFI (median fluorescence intensity) and the red lines show the median values. **** *p* < 0.0001, *** *p* < 0.001, ** *p* < 0.01, and * *p* < 0.05.

**Figure 5:**
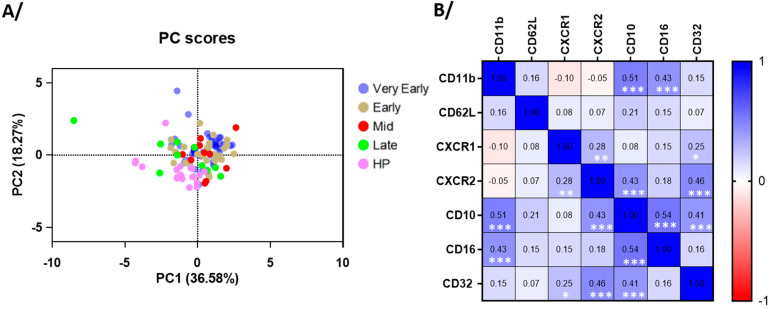
The principle component and correlative analyses of the level of surface membrane proteins on blood neutrophils. (**A**) The principal component analysis of the participants divided as healthy participants (HPs), very early phase, early phase, mid-phase, and late phase COVID-19. (**B**) The correlative analysis (Spearman) of all surface markers and timepoints in COVID-19 (*n* = 90). *r* values are shown, *** *p* < 0.001, ** *p* < 0.01, and * *p* < 0.05.

The surface level of CD11b and CD62L, two markers of activated or primed neutrophils [21], did not change at any timepoints during hospitalization compared to healthy participants (Figure 4A). However, reductions in CXCR1 and CXCR2 on the neutrophil surface, particularly in the early phases (Figure 4A), may be an indication of interactions with their ligands and chemotaxis toward the tissue [22]. Analyses of neutrophil maturity markers showed an increased proportion of blood immature neutrophils characterized by a lower CD10 expression and an enhanced percentage of CD10/CD16^low^ and CD10^low^CD177^high^ neutrophils (Figure 4). Furthermore, activated (CD11b^high^CD62L^low^) and immunosuppressive (CD16^high^CD62L^low^) neutrophil subpopulations were significantly increased (Figure 4B). Notably, the changes in neutrophil populations were mainly observed during the early phase and mid-phase rather than during the very early and late phases (Figure 4B), suggesting a temporal evolution of neutrophil responses during COVID-19 progression. Note that the PCA (Figure 5A) of the seven surface markers shown in Figure 5B did not differentiate COVID-19 patients from healthy individuals as distinctively as for the eosinophils in Figure 3A (Figure 5A). In agreement with data in Figure 4, and the immature neutrophil profile during COVID-19, CD10 correlated with CD16 as well as CXCR2 (Figure 5B). In addition, CD10 was strongly correlated with both CD11b and CD32, another marker of activation, strengthening the enrichment of an immature and activated blood neutrophil subpopulation in COVID-19 (Figure 5B).

### 3.5. Blood Monocyte Phenotype

**Figure 6:**
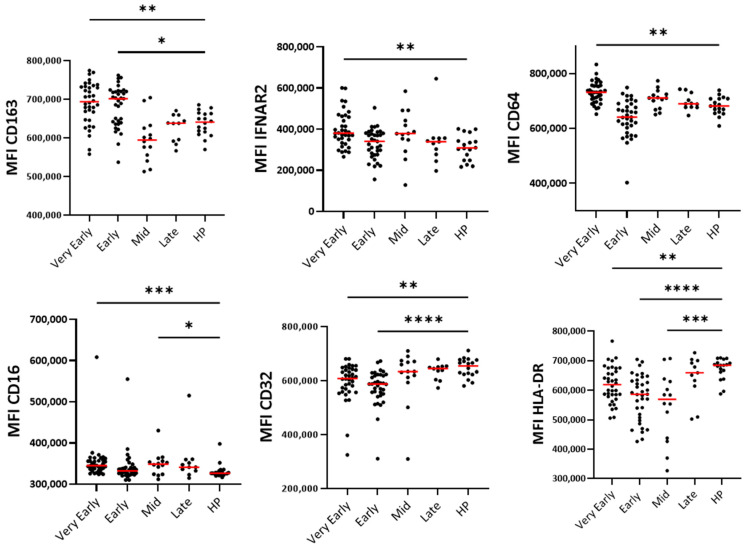
Level of surface proteins on blood monocytes over time after hospitalization with SARS-CoV-2 infection and compared to healthy controls. As shown in Table 2, timepoints were grouped as “very early” phase (D1 to D3), “early” phase (D5 to D9), “mid” phase (D14), and “late” phase (D30). MFI (median fluorescence intensity) and the red lines show the median values. **** *p* < 0.0001, *** *p* < 0.001, ** *p* < 0.01, and * *p* < 0.05.

**Figure 7:**
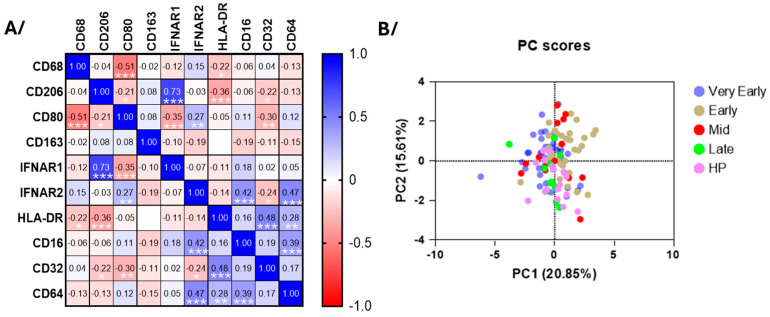
The principle component and correlative analyses of the level of surface membrane proteins on blood monocytes. (**A**) The correlative analysis (Spearman) of all surface markers and timepoints in COVID-19 (*n* = 96). *r* values are shown, *** *p* < 0.001, ** *p* < 0.01, and * *p* < 0.05. (**B**) The principal component analysis of the participants divided as healthy participants (HPs), very early phase, early phase, mid-phase, and late phase COVID-19.

The surface monocyte CD64, CD16, and IFNAR2 were all higher in the very early phase of COVID-19 (Figure 6) and did correlate with each other (Figure 7A). During both early phases, monocytes displayed high levels of CD163, while CD32 and HLA-DR were decreased in the early phases and mid-phase (Figure 6). CD32 and IFNRA2 correlated with HLA-DR and CD206, respectively, but neither CD206, CD80, CD68, or IFNAR1 showed changes at any timepoints during hospitalization (Figure 6 and Figure 7 and Supplementary Figure S5). As for the neutrophils, the PCA using all surface markers at all timepoints did not differentiate COVID-19 patients from healthy individuals as distinctively as for the eosinophils (Figure 7B).

### 3.6. Measurement of Cytokines, Growth Factors, and Markers of Cell Activation in Serum

**Figure 8:**
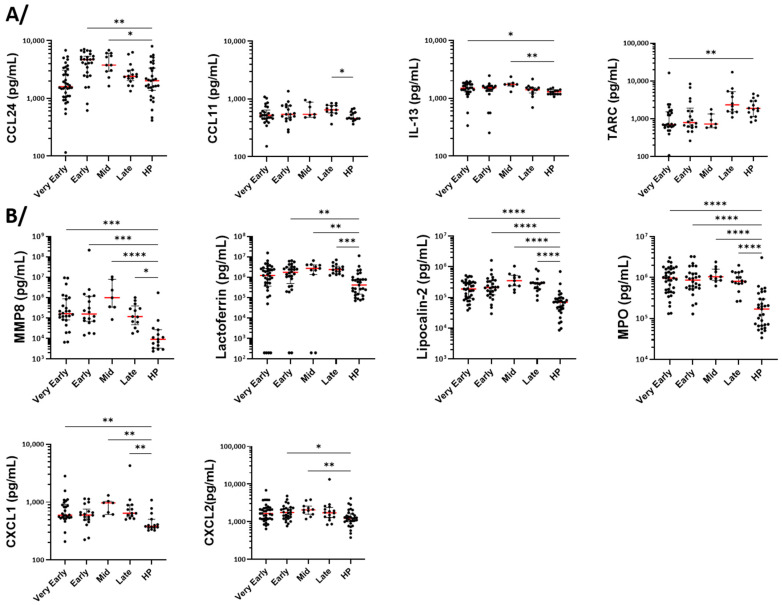
The quantification of serum cytokines, chemokines, and growth factors related to eosinophils and neutrophils. Mediators were quantified by multiplex assays at different periods (phases) after the hospitalization of COVID-19 patients and at one timepoint in a total of 31 healthy individuals. (**A**) The type-2 (T2) immune response and pro-eosinophilic mediators. (**B**) Mediators related to neutrophil activation and chemotaxis. The red lines show the median values. **** *p* < 0.0001, *** *p* < 0.001, ** *p* < 0.01, and * *p* < 0.05. The Kruskal–Wallis test with Dunn’s multiple comparisons test.

We used the serum from 37 and 28 patients in the very early and early period, respectively (Table 2). The number of sera was then reduced from the mid-phase for the same reasons as indicated above when describing flow cytometry (flow chart in Figure S4). To the 18 healthy individuals used for flow cytometry, sera from 13 other healthy participants were added for the measurement of mediators by Luminex (Table 2). Among the type-2 (T2) and eosinophilic mediators, eotaxin-2 (CCL24) was augmented in the both early (D5–D9) and mid-phase (D14) compared to controls, while eotaxin-1 (CCL11) was elevated in the late phase only (Figure 8A). IL-13 increased in both the very early and mid-phase, while TARC was lower, particularly at the very early phase, before returning to a normal level in the late phase (Figure 8A). This pattern indicates the chronological regulation of different chemotactic signals for eosinophils and cells of the type-2 (T2) immune response during the COVID-19 progression. None of the other measured mediators related to eosinophils and the T2 response (CCL26, IL-3, IL-4, IL-5, CD44, TSLP, or GM-CSF) displayed any significant differences between the different timepoints and between COVID-19 versus controls (Supplementary Figure S6).

Regarding the mediators related to neutrophilic activity, MMP8, lactoferrin, lipocalin-2, and MPO (all proteins potentially released by activated neutrophils) were augmented in the serum at all timepoints during COVID-19 (Figure 8B). CXCL1 and CXCL2 were also enhanced at most timepoints, suggesting an increased neutrophil activation and recruitment (Figure 8B). Proteinase 3 was also increased but displayed a very high heterogeneity among patients. Although statistically significant at the early phase, only small changes were seen for IL-8 (Supplementary Figure S6).

**Figure 9:**
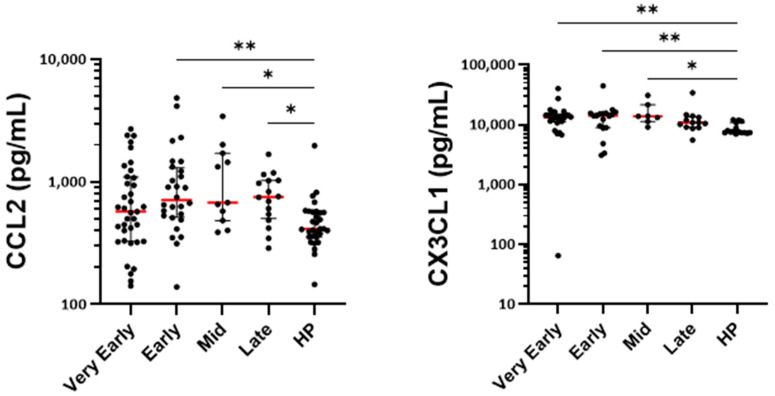
The quantification of serum chemokines related to monocytes. Mediators were quantified by multiplex assays at different periods (phases) after the hospitalization of COVID-19 patients and at one timepoint in a total of 31 healthy individuals (HP). ** *p* < 0.01, and * *p* < 0.05. The Kruskal–Wallis test and Dunn’s multiple comparisons test.

Among the other chemokines measured in the serum, CX3CL1 was elevated in the early phases and the mid-phase, suggesting a possible rapid enhanced recruitment of non-classical monocytes. In contrast, CCL2, a chemoattractant for classical monocytes, was augmented only at later timepoints (Figure 9), indicating the sequential recruitment of different monocyte subpopulations.

## 4. Discussion

This study followed the evolution of the blood innate immune cells count and phenotype over time during COVID-19, particularly in the most severely affected patients that required hospitalization. Distinctly from previous studies that had used one or two timepoints during the disease, the present kinetics allow us to better comprehend changes during the disease. We found that while blood eosinophil counts quickly and dramatically dropped after the infection with SARS-CoV-2, their phenotype changed into CD69/CD125/CD63^high^ and CD44/CCR3^low^. The blood monocytes count was also lower on the first day of hospitalization when their phenotype was CD64/CD16/IFNAR2^high^. Unlike eosinophils and monocytes, the number of neutrophils did not change significantly during the very early days but rather after 5 days of hospitalization, when the proportion of immature (CD10/CD16^low^ and CD10^low^CD177^high^) and activated (CD11b^high^CD62L^low^) blood neutrophils was also augmented the most significantly.

### 4.1. Eosinopenia and Change in Phenotype

Our data agrees with previous observations showing eosinopenia during COVID-19 [1,2,3,4,5]. Surprisingly, the quick suppression of blood eosinophil numbers was not concomitant to a significant augmentation of blood eotaxins nor any striking changes in T2 markers, suggesting neither eotaxins-driven eosinophil chemotaxis to the tissue nor a lack of eosinophil differentiation due to reduced IL-5 may be the causes of eosinopenia. In fact, concerning the latter, previous studies have shown that IL-5, the major cytokine acting on eosinophil differentiation, was rather increased in COVID-19 compared to healthy individuals [23,24]. In addition, our group has previously shown that the PBMC from patients with COVID-19 activated with mixed antigens from SARS-CoV-2 produce more IL-5 in the severe COVID-19 population [23]. Regarding eosinophil chemotaxis, other studies have demonstrated that the migration of eosinophils toward the infected tissue during COVID-19 particularly enhanced numbers of eosinophils in the bronchoalveolar lavage (BAL), likely and partially due to an increase in BAL CCL11 (eotaxin-1) [24]. Also, the analyses of post-mortem RNA-sequencing with COVID-19-infected lungs versus normal controls show the significant expression and upregulation of genes related to eosinophilia, such as *CLC* (Galectin-10), *RNASE2* (EDN), and *CCL11* (eotaxin-1) [25]. Therefore, although we did not observe major early increases in blood eotaxins, eotaxins, and/or other chemokine receptors, ligands must be produced in tissues to trigger the important recruitment of eosinophils into the lung, explaining the occurrence of a “very early” important eosinopenia in COVID-19.

The augmentation of surface CD69 and the reduction in CCR3 are markers of activated mature eosinophils by the beta-chain ligands (IL-3, IL-5, and GM-CSF) in vitro [26,27]. However, unlike what is typically seen with activated mature blood eosinophils, we found here increased CD125 and CD63, a reduction in CD44, and a lack of change for HLA-DR and CRTH2 in COVID-19. In addition, the blood eosinophil phenotype in COVID-19 did not correspond either to a tissue eosinophil phenotype, such as in allergic asthma where activated airway eosinophils displayed a very low amount of surface CD125 and high amounts of CD44 [28]. This suggests that in our hospitalized patients with COVID-19, eosinophils have not been activated after maturation when present in circulating blood, but blood eosinophils rather leave the bone marrow differentially differentiated and matured during COVID-19. In addition, although receptors involved in chemotaxis, such as CRTH2 or CCR3, were not changed or were only slightly reduced, we, however, cannot totally rule out that blood eosinophils during COVID-19 represent a small and unique population that have not been recruited into the tissue. Notably, in a T2 disease such as atopic dermatitis, we recently identified a very different blood eosinophil phenotype compared to COVID-19. In fact, in atopic dermatitis, eosinophils displayed no change in surface CD125, which also did not correlate with CD69 but was associated instead with CD44 and HLA-DR [18]. The origins and the differential functions of eosinophils with a phenotype characterized as CD69/CD125/CD63^high^ and CD44/CCR3^low^ could be subjects of further investigations, particularly in other viral respiratory diseases, such as influenza or respiratory syncytial virus infection.

### 4.2. Neutrophilia and Change in Phenotype

We observed a slight increase in the neutrophil count starting at D7 after hospitalization. This observation supports previously reported changes in neutrophil counts during COVID-19 [3,4,8,9], but the lack of concomitance with eosinopenia does not support the fact that eosinopenia was due to the reprogramming of the granulocyte precursor toward neutrophils in the bone marrow. However, an elevated neutrophils count occurred following a slight augmentation of blood IL-8, a key chemokine for neutrophils, which is associated with the recruitment of immunosuppressive cells [29] and NETosis-prone neutrophils. This agrees with the enrichment in the immunosuppressive neutrophil population (i.e., CD16^high^CD62L^low^) found in our study. In addition, early increased serum CXCL1 and CXCL2 in most timepoints and reduced amounts of surface C-X-C chemokine receptor type 1 (CXCR1) and CXCR2 suggest that a subset of neutrophils migrates to the tissue.

Interestingly, we did not find any significant change in surface CD62L or CD11b on the neutrophil population as reported during acute COVID-19 [10]. Yet, we identified clear changes in neutrophil subpopulations when using two surface markers. Therefore, from the fifth day of hospitalization, blood neutrophils start displaying a clear immature, immunosuppressive, and activated phenotype, i.e., CD10/CD16^low^ and CD10^low^CD177^high^, CD16^high^CD62L^low^, and CD11b^high^CD62L^low^, respectively. These neutrophil populations may limit the spread of infection [30] but may also be more prone to NETosis with a possible role in pulmonary embolisms [13]. The activated state of blood neutrophils as defined by the enhanced CD11b^high^CD62L^low^ neutrophil population was supported by the elevated serum MMP8, MPO, lipocalin-2, and lactoferrin at almost all timepoints after hospitalization, including the late phase of hospitalization. Thus, although these data do not rule out a very early recruitment of neutrophils during COVID-19, they suggest a more delayed and prolonged implication of neutrophils compared to eosinophils, with potential longer damaging effects of immature and immunosuppressive neutrophils.

### 4.3. Monocytes

Although the decrease in the blood monocyte number was only seen at the earlier timepoint, our study agrees with a previous report where monocytes decreased in the peripheral blood within a week after the onset of the disease, signifying the efflux of monocytes from the blood to the pulmonary site [31]. Furthermore, it has been previously proposed that there is an important migration of both inflammatory transitional and non-classical monocytes to the lung during severe COVID-19, leading to lung damage [7,14]. This corroborates with our data where, contrary to CCL2, serum CX3CL1 was quicky augmented suggesting that non-classical monocytes (NC; CD14+/-CD16++/CX3CR1+) rather than classic monocytes (CL; CD14++CD16−/CCR2+) are the first actors during COVID-19. Supporting a role for NC monocytes, patients with a serious COVID-19 trajectory displayed a decreased number of blood NC [15]. Of note, in severe COVID-19 patients, lung tissue monocyte-derived macrophages may recruit neutrophils via the production of ligands for CXCR2 [32]. Therefore, the diminution of blood monocytes was likely the consequence of the monocyte migration into tissue and was possibly a prelude to the recruitment and activation of neutrophils.

In accord with a previous study that reported dysregulated and immature CD163^high^ and HLA-DR^low^ blood monocytes in severe COVID-19 but not in healthy controls [33], we found elevated CD163 and lower HLA-DR on the surface of monocytes in our study. Furthermore, the high-affinity receptor for IgG (CD64) as well as the interferon-α/β receptor (IFNAR2) were both enhanced at the very early phase after hospitalization, and they were globally strongly correlated over time. We may speculate that the engagement of CD64 with IgG immune complexes can lead to a monocyte infection causing cell death and systemic inflammation contributing to the COVID-19 pathogenesis [34], whereas type I interferons can activate inflammatory monocytes to develop an early natural killer antiviral response [35].

### 4.4. General Roles of the Innate Cells in Viral Infections and Limitations of the Study

The potential contribution of varying leukocytes, including acute inflammation and cytokine release, in COVID-19 and other respiratory virus infections has been widely reported. Monocytes amplify inflammation through the NF-κB-driven cytokine release and inflammasome activation [33,36]. Neutrophils promote tissue injury via ROS, proteases, and NET formation [37,38]. In contrast, eosinophils can exert protective antiviral functions through nitric oxide release, degranulation, and promoting T cell responses [39,40,41,42]. Although the work presented here begins to delineate specific roles in the COVID-19 pathogenesis, it represents only the initial step in what is expected to become a broader and more comprehensive line of investigation. Our study has several limitations. First, by design, it focused only on hospitalized patients with respiratory symptoms, limiting the extrapolation to milder or asymptomatic cases. Second, innate immune cells could not be analyzed in the lung tissue, and functional assays could not be performed due to limited access to patients’ samples and technical constraints during the peak pandemic period. Also, the number of patients was unfortunately reduced at some timeframes due to the reasons indicated above in the manuscript. This reduction in the number of patients did not allow us to examine the immune cell phenotypes in relation to the clinical outcomes of the disease, which could have been ineffective anyway due to the overall severity of the enrolled patients. In addition, we did not compare SARS-CoV-2 infections with other viral respiratory infections to determine if our results are specific to COVID-19. These aspects merit future investigation.

## 5. Conclusions

Our present study examined the evolution of the blood innate cells number and phenotype over a relatively long period during the hospitalization of patients with COVID-19. We found a dramatic and rapid reduction in the blood eosinophil count accompanied by a noticeable change in the blood eosinophil phenotype, including enhanced surface CD69, the main marker of eosinophil activation, and CD125, the receptor for IL-5. The phenotypic profile does not match a profile defining an activated mature blood eosinophil and thus suggests differential programming in the bone marrow. Conversely, the increase in the neutrophil number was settled and delayed as were the changes in surface markers. Yet, the presence of immature, immunosuppressive, and activated blood neutrophils remained elevated for at least 14 days and up to 30 days after hospitalization with the potential of damaging lung tissues. Our data also point out that non-classical monocytes may have a very early role to control the infection. The role of monocytes/macrophages on the neutrophil activation during COVID-19 has already been depicted, but the role of eosinophils in this process remains unknown. Finally, the mediators responsible for the changes in innate immune cell phenotypes during COVID-19 remain unidentified.

## Figures and Tables

**Table 1:** Characteristics of Participants Used for Immune Cells Phenotyping.

		Patients with SARS-CoV-2 Infection	Healthy Participants
Number of enrolled participants (n)		40	18
Age; median (years) [Min–Max]		63 [41–99]	71 [53–89]
Sex; Males/Females (n) [% Males]		28/9 [75%]	10/8 [55%]
Deceased (n)		5	
Severity status (WHO scale); median [Min–Max]		5 [4–8]	
Symptoms	Fever	24 [60%]	
	Cough	24 [60%]	
	Dyspnea	32 [80%]	
Treatments	Antibiotics	26 [65%]	
	Antivirals	12 [30%]	
	Corticosteroids	30 [75%]	

Patients’ symptoms and treatments during the 30 days of hospitalization after infection with SARS-CoV-2.

**Table 2:** Number of Participants of Each Analysis and at Each Timeframe.

Participants	Eosinophils (Flow Cytometry)	Neutrophils (Flow Cytometry)	Monocytes (Flow Cytometry)	Mediators in Sera (Luminex)
Patients COVID-19				
Very early phase (D1 to D3)	13 (10 for antibody panel 2)	37	36	37
Early phase (D5 to D9)	27 (23 for antibody panel 2)	33	35	28
Mid-phase (D14)	13	10	14	11
Late Phase (D30)	10	10	11	16
Healthy Particpants	18	18	18	31

## Data Availability

The datasets generated and analyzed during the current study are available from the corresponding authors on reasonable request.

## Supplementary Materials

The following supporting information can be downloaded at https://www.mdpi.com/article/10.3390/cells14141093/s1.

## Abbreviations

The following abbreviations are used in this manuscript:

COVID-19	coronavirus disease 2019
D1	day 1
MFI	median fluorescence intensity
NETosis	neutrophil extracellular traps formation
PCA	principal component analysis
SARS	severe acute respiratory syndrome
T2	type-2
