# Supplementary material for: Evolution of Blood Innate Immune Cell Phenotypes Following SARS-CoV-2 Infection in Hospitalized Patients with COVID-19
Source: Cells. 2025 Jul 17;14(14):1093. doi: 10.3390/cells14141093 (PMC12293952; doi:10.3390/cells14141093)
Supplement: Supplementary file 1 [file cells-14-01093-s001.zip › cells-3668595-supplementary.pdf]

Supplementary Figures and Tables

Supplementary Table S1. Antibody panels used to phenotype immune cells by flow cytometry

	Analyte	Fluorochrome	Manufacturer	Cat#
**Panel 1**	CD63	FITC	Beckman Coulter	B92467
**Eosinophils**	CD193	APC	Miltenyi	130-123-300
	CD16	Pacific Blue	Beckman Coulter	B36292
	CD45	Krome-Orange	Beckman Coulter	B36294
	CD125	PE	BD	555902
	CRTH2	PE-Vio770	Miltenyi	130-119-624
**Panel 2**	CD44	FITC	Beckman Coulter	IM1219U
**Eosinophils**	CD69	APC	Beckman Coulter	A80711
	CD16	Pacific Blue	Beckman Coulter	B36292
	CD45	Krome-Orange	Beckman Coulter	B36294
	HLA-DR	PE	BD	555812
**Panel 3**	CD10	PC7	Beckman	B96750
**Neutrophils**	CD11b	APC	Beckman	A87782
	CD62L	APC-A750	Beckman	B26604
	CD15	AF 700	Biolegend	301920
	CD16	Pac blue	Beckman	B36292
	CD19	PC5.5	Beckman	B49211
	CD45	Krome Orange	Beckman	B36374
	CRTH2	Pe/Dazzle 594	Biolegend	350126
	CXCR1	FITC	eBioscience	11-1819-42
	CXCR2	PE	eBioscience	12-1829-42
	CD177	BV650	BD	745366
	CD32	BV605	BD	740399
**Panel 4**	CD64	ECD	Beckman	A98434
**Monocytes**	CD80	APC-A750	Beckman	B30643
	IFNAR1	APC	R&D systems	FAB245A
	IFNAR2	PE	Miltenyi	130-099-555
	CD14	PC5.5	Beckman	A70204
	CD16	Pac blue	Beckman	B36292
	CD206	AF 700	Biolegend	321132
	CD45	Krome Orange	Beckman	B36374
	CD68	PC7	Biolegend	333816
	CD163	FITC	Miltenyi	130-112-132
	CD32	BV605	BD	740399
	HLA-DR	BV650	BD	564231

Supplementary Figure S1

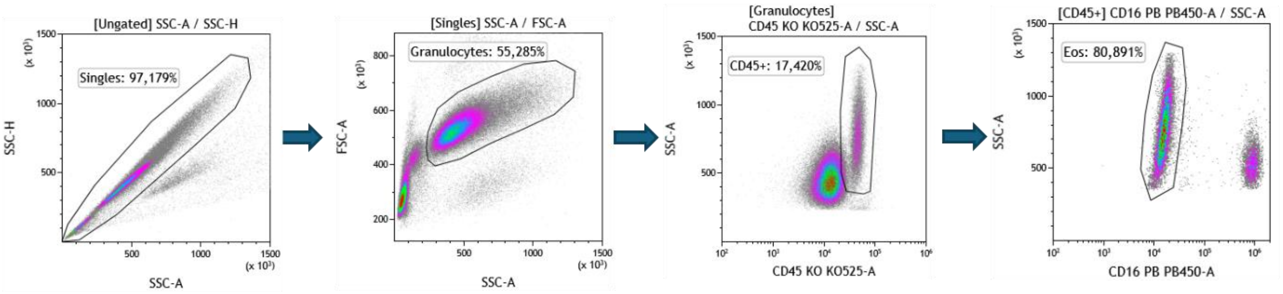

Supplementary Figure E1: Gating strategy for identifying eosinophils in blood samples using flow cytometry. Doublets cells were first excluded, and granulocytes were selected based on FSC and SSC. CD45^{+} cells were selected to focus on leukocytes, and then eosinophils were identified by gating on CD16^{negative} cells.

Supplementary Figure S2

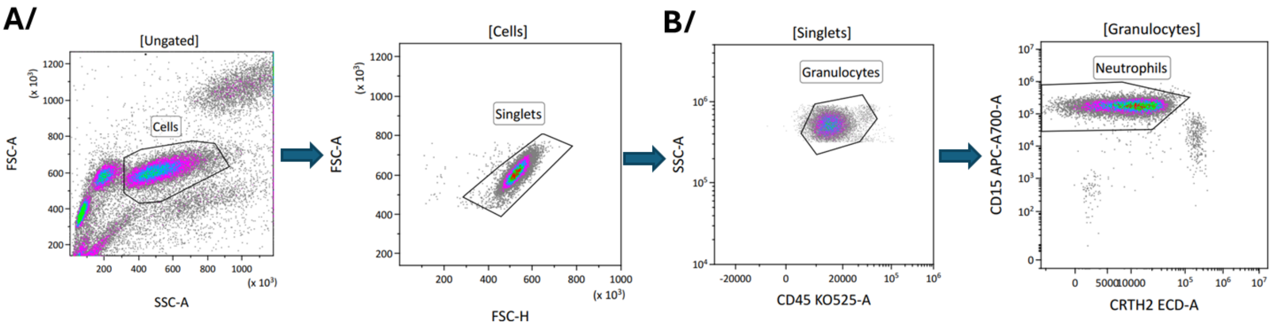

Supplementary Figure E2. Gating strategy for neutrophils using flow cytometry. A/ Cells of interest were gated using FSC-A/SSC-A, and single cells were gated using FSC-A/FSC-H. **B/** Granulocytes were gated using SSC-A and CD45^{positive}, and then neutrophils were gated using CD15^{positive}/CRTH2^{negative} .

Supplementary Figure S3

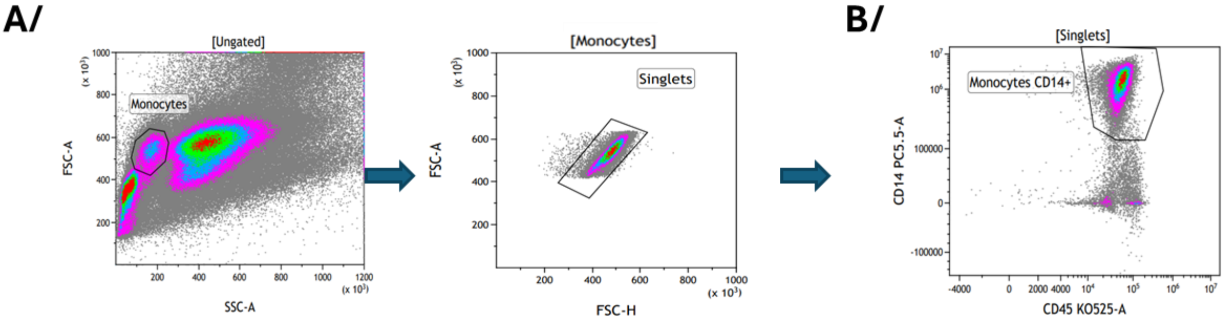

Supplementary Figure E3. Gating strategy for monocytes using flow cytometry. A/ Cells of interest were gated using FSC-A/SSC-A, and single cells were gated using FSC-A/FSC-H. **B/** Monocytes were then gated using CD14 ^{positive}/CD45^{positive}.

Supplementary Figure S4. Flow chart explaining the number of patients included at each timeframe after hospitalization for eosinophil phenotyping by flow cytometry

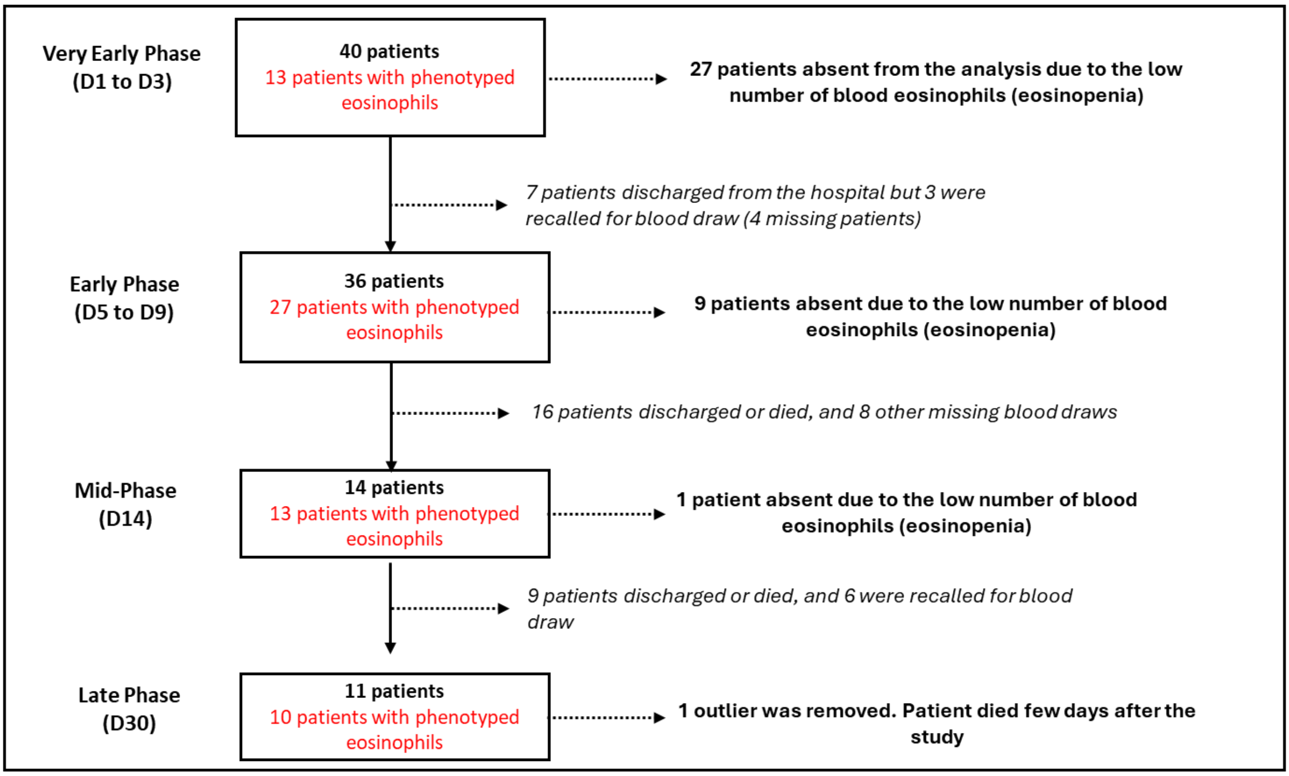

Supplementary Figure S5

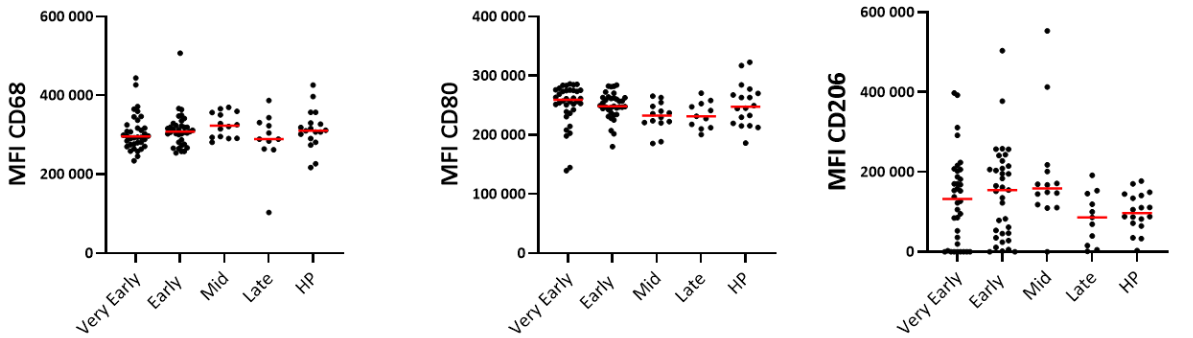

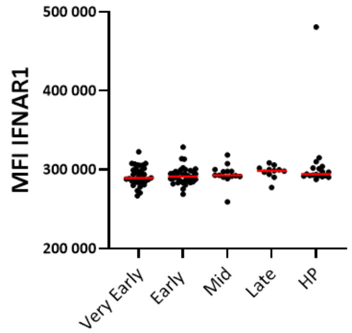

Supplementary Figure E5. Level of surface proteins on blood monocytes overtime after hospitalization with SARS-Cov-2 infection and compared to healthy controls. As shown in Table 2, timepoints were grouped as "Very Early" phase (D1 to D3), "Early" phase (D5 to D9), "Mid" phase (D14), and "Late" phase (D30). MFI (median fluorescence intensity). No statistically significant differences between timepoints.

Supplementary Figure S6

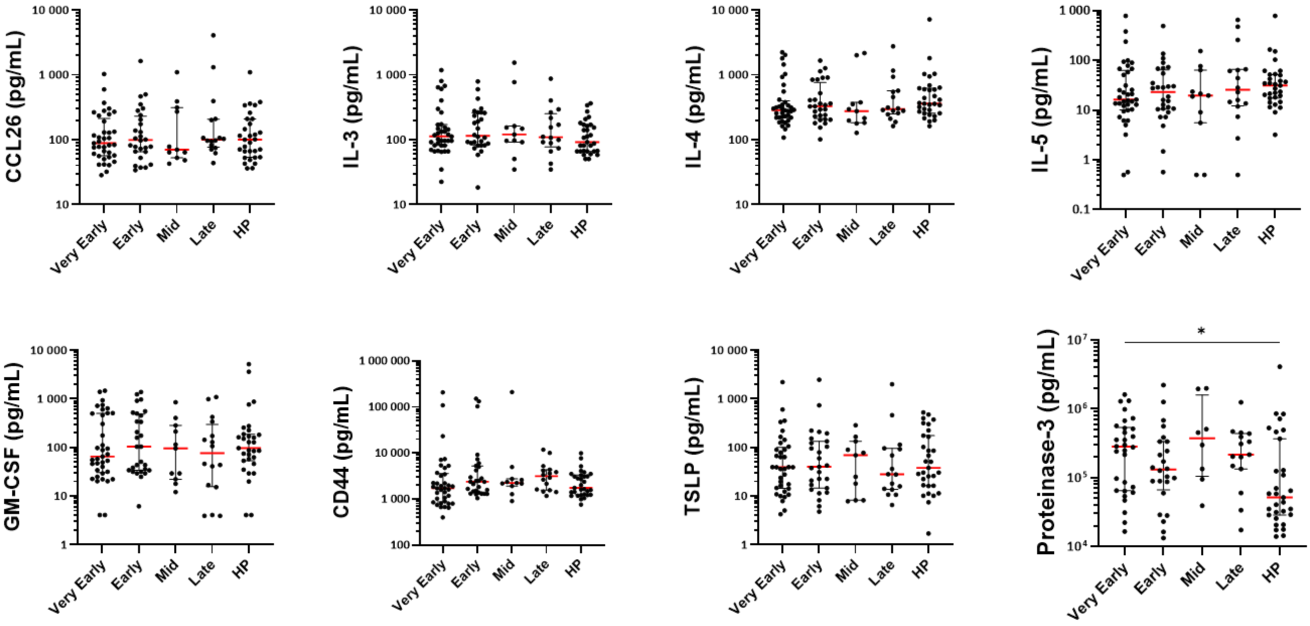

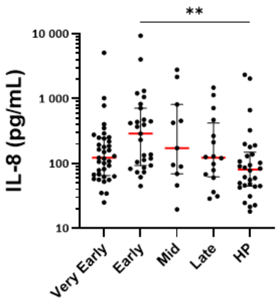

Supplementary Figure E6. Quantification of serum cytokines, chemokines and growth factors related to eosinophils and neutrophils. Mediators were quantified by multiplex assays at different periods (phases) after hospitalization of COVID-19 patients and at one timepoint in a total of 31 healthy participants (HP). **p<0.01, *p<0.05. Kruskal-Wallis test with Dunn's multiple comparisons test.
